# Dietary *Clostridium butyricum* and *Bacillus subtilis* Promote Goose Growth by Improving Intestinal Structure and Function, Antioxidative Capacity and Microbial Composition

**DOI:** 10.3390/ani11113174

**Published:** 2021-11-06

**Authors:** Jie Yu, Biao Dong, Minmeng Zhao, Long Liu, Tuoyu Geng, Daoqing Gong, Jian Wang

**Affiliations:** 1 College of Animal Science and Technology, Yangzhou University, Yangzhou 225009, China; yuj126315@163.com (J.Y.); mmzhao@yzu.edu.cn (M.Z.); liujiaolong688@sina.com (L.L.); tygeng@yzu.edu.cn (T.G.); 2 Department of Animal Science and Technology, Jiangsu Agri-Animal Husbandry Vocational College, Taizhou 225300, China; dongbiao8201@126.com

**Keywords:** *Bacillus subtilis*, *Clostridium butyricum*, geese, gut microbiota, production performance

## Abstract

**Simple Summary:**

In this study, the effects of dietary supplementation of *Clostridium butyricum* and/or *Bacillus subtilis* were determined on growth performance, intestinal antioxidative capacity, intestinal morphology, cytokine production, and intestinal microbial composition in Yangzhou geese. Data showed that probiotics promoted feed intake and growth, improved antioxidative capacity and intestinal morphology, increased the relative abundances of *Firmicutes* and *Lactobacillus* in intestinal content, decreased the relative abundances of *Proteobacteria* and *Ralstonia*, and altered α-diversity and the predicted functions of intestinal microflora, but did not induce the expression of genes related to intestinal inflammation and tight junction.

**Abstract:**

Probiotics are a substitute for antibiotics in the sense of intestinal health maintenance. *Clostridium butyricum* and *Bacillus subtilis*, as probiotic bacteria, have been widely used in animal production. The aim of this study was to investigate the effects of the two probiotic bacteria in geese. A total of 288 1-day old, healthy Yangzhou geese were randomly assigned into 4 groups (A, B, C and D) with 6 replicates of 12 birds each. Group A, as control, was fed a basal diet, and the treatment groups (B, C and D) were fed the basal diet supplemented with 250 mg/kg *Clostridium butyricum* (the viable count was 3.0 × 10^6^ CFU/g), 250 mg/kg *Bacillus subtilis* (the viable count was 2.0 × 10^7^ CFU/g), or a combination of the two probiotic bacteria for 70 days, respectively. The results indicated that: compared with the control group, dietary probiotics (1) promoted the growth and feed intake of the geese, (2) increased the absolute weight of duodenum, (3) increased the antioxidative capacity (total antioxidative capacity (T-AOC), total superoxide dismutase (T-SOD) and glutathione peroxidase (GSH-PX)) of intestinal mucosa, (4) improved intestinal morphology (the ratio of villus height to crypt depth), (5) but did not induce inflammation and changes of tight junction in the intestine, which was indicated by no induction of pro/inflammatory cytokines (*IL-1β*, *IL-6*, *IL-10*, *TNFAIP3*) and tight junction related genes (*TJP1* and *OCLN*). Moreover, dietary probiotics increased the relative abundances of *Firmicutes* phylum and *Lactobacillus* genus and decreased the relative abundances of *Proteobacteria* phylum or *Ralstonia* genus in the intestinal content. In addition, the alpha diversity (observed species, Chao1, and estimate the number of OTUs in the community(ACE)) was reduced and the predicted functions of intestinal microflora, including peptidases, carbon fixation and metabolic function of starch and sugar, were enhanced by dietary probiotics. In conclusion, dietary probiotics promote the growth of geese by their positive effects on intestinal structure and function, the composition and functions of gut microflora, and intestinal antioxidative capacity.

## 1. Introduction

The extensive use of antibiotics in animal production has led to many problems such as superbacteria and drug residues, and these problems make the producers and consumers concerned about the safety of livestock and poultry products. Probiotics are popular substitutes for antibiotics because of their high efficiency and safety.

Probiotics can prevent imbalance of gut microbiota, enhance intestinal barrier function, and regulate cytokine production [1]. Previous studies indicate that the performance of livestock is closely related to intestinal microbial load, the morphology and structure of the intestinal wall, and the activity of the immune system [2]. *Clostridium butyricum*, a beneficial bacterial species widely colonized in animal intestines, can promote growth, improve immunity, and regulate intestinal microbial composition in host animals [3]. In addition, *Clostridium butyricum* has a strong resistance to environmental changes. *Clostridium butyricum*, therefore, is a good choice for probiotics. Similarly, *Bacillus subtilis* can also serve as a non-toxic, non-residual and non-drug-resistant probiotic product. It has been demonstrated that *Bacillus subtilis* can maintain the balance of gut microbiota and the integrity of intestinal mucosal barrier, regulate nutrient metabolism, and strengthen animal immunity [4].

Although single probiotic bacteria, such as *Clostridium butyricum* and *Bacillus subtilis*, have been widely applied in animal production, the effect of compound probiotics is less investigated. There are only several studies on the effects of multistrain probiotics performed in poultry. For example, Zhang et al. investigated the efficacy of *Lactobacillus acidophilus, Bacillus subtilis*, and *Clostridium butyricum* supplementation in broilers, and the data indicated that dietary supplementation with multistrain probiotics improved broiler growth performance, ileal amino acids digestibility, and humoral immunity, but decreased the cecal numbers of E. coli and the NH3 content of excreta [5]. Zeng et al. also demonstrated that supplementation of compound probiotics (*Clostridium butyricum*-, *Bacillus subtilis*-, and *Bacillus licheniformis*) to diet improved the growth performance, serum immune responses, the ratio of ileal villus height to crypt depth, major caecal short-chain fatty acids(SCFAs), and overall health in broiler chickens [6]. Multistrain probiotics could be superior to single probiotics as different species of probiotic bacteria may promote animal health and production performance through different mechanisms, such as the release of different metabolites, different effects on gut microbial composition, and cooperative action between different bacterial species in compound probiotics [7]. On the other hand, there may be antagonism among different bacterial species in compound probiotics. These putative mechanisms, however, are not validated yet. Compared with chickens, the application of single or combinational probiotics in the goose industry is rarely reported.

In this study, the effects of dietary supplementation of *Clostridium butyricum, Bacillus subtilis* or their compound were determined on growth performance, intestinal antioxidative capacity, intestinal morphology, cytokine production, and intestinal microbial composition in Yangzhou geese. The results may provide some guidelines for use of *Clostridium butyricum* and *Bacillus subtilis* as probiotics in goose production.

## 2. Materials and Methods

### 2.1. Experimental Design

The animal experiment protocol in this study complied with relevant guidelines for animal welfare and use, and has been approved by the Yangzhou University Animal Ethics Committee (the certificate number authorized by IACUC is SYXK(Su)2016-0020). A total of 288 1-day-old, healthy Yangzhou male geese from the same batch were randomly assigned to 4 groups (control group A, *Clostridium butyricum* group B, *Bacillus subtilis* group C, and multi-species probiotics group D) with 6 replicates per group and 12 geese per replicate. The experiment lasted for 70 days. Geese in group A, as control, were fed a basal diet, and those in the treatment groups (the groups B, C and D) were fed the basal diet supplemented with 250 mg/kg *Clostridium butyricum* (viable count 3.0 × 10^6^ CFU/g), 250 mg/kg *Bacillus subtilis* (viable count 2.0 × 10^7^ CFU/g), or the combination of 250 mg/kg *Clostridium butyricum* and 250 mg/kg *Bacillus subtilis*, respectively. *Clostridium butyricum* and *Bacillus subtilis* were provided by Suzhou Kapaleisen Biotechnology Co., Ltd(Suzhou, China).

The basal diet was formulated according to poultry nutritional requirements issued by the Nutritional Research Committee (1994), and its ingredient and nutritional compositions are shown in Table S1. The geese were raised in the National Waterfowl Gene Bank (Taizhou, Jiangsu, China). The house and facilities were first cleaned and disinfected, followed by allocating the experimental goslings to cages with 12 geese per cage during day 1 to day 28 of age. The geese were then transferred to individual cages and reared for the remaining days (day 29 to day 70 of age). Natural light and routine husbandry management were applied during the experiment period. All the geese had free access to diet and water.

### 2.2. Phenotypic Measurement and Sample Collection

The feed intake was measured weekly. The body weight of each goose was weighed at the age of 70 days after 12 h of fasting. The average daily gain (ADG) and the average daily feed intake (ADFI) were calculated for each group during day 1 to day 70 of age. Two geese with body weights close to the average body weight were selected from each replicate for sacrifice. The weights and lengths of different intestinal segments (duodenum, jejunum, ileum and cecum) were measured.

Mucosal samples were gently scraped from the jejuna and ilea of all the experimental geese with a glass slide and stored in liquid nitrogen. Total antioxidative capacity (T-AOC), total superoxide dismutase (T-SOD), and glutathione peroxidase (GSH-PX) activities of mucosal samples were measured according to the manufacturer’s instructions using the kits for total antioxidative capacity, total superoxide dismutase, and glutathione peroxidase (Jiancheng Bioengineering Institute, Nanjing, China).

For histomorphological analysis, about 0.5 cm of jejunum and ileum tissues were cut and placed in formaldehyde fixative solution, followed by dehydrating in xylene and ethanol and embedding in paraffin wax. The jejunum and ileum tissue sections (5 μm) were then made and stained with hematoxylin and eosin (HE). Using the tissue sections, villus length and crypt depth of jejunum and ileum were determined with MShot Image Analysis System software under a microscope. More than 10 sites on each section were measured for jejunal or ileal tissues (2 sections per replicate).

Lastly, the middle parts of the jejunum and ileum tissues of each goose (2 cm long) were collected, snap-frozen in liquid nitrogen, and stored at −80 ℃ for later use. The contents of the jejunum and ileum were also collected for 16S rDNA sequencing analysis of gut microbiota.

### 2.3. Quantitative PCR Analysis

Total RNA was extracted from intestinal tissues with TAKARA’s RNAiso Plus total RNA extraction kit (TaKaRa Biotechnology Co. Ltd., Dalian, China), according to the manufacturer’s instructions. The concentration of RNA was determined by a spectrophotometer, and the quality of the RNA was determined by the ratio of absorbance at 260 nm to 280 nm. Reverse transcription was performed with the Hisscript^®^ Q RT Supermix(Vazyme Biotech Co., Ltd, Nanjing, China) for qPCR reverse transcription kit, according to the manufacturer’s instructions. The relative mRNA expression of the selected genes was determined by quantitative real-time polymerase chain reaction (qPCR). The primers of target and reference genes are shown in Table 1. The relative expression level of each target gene was calculated by 2^−∆∆Ct^ method.

### 2.4. 16S rDNA Sequencing Analysis

The genomic DNA was extracted from intestinal contents by cetyl trimethyl ammonium bromide (CTAB) or sodium dodecyl sulfonate (SDS) methods. The purity and concentration of the DNA samples were examined by agarose gel electrophoresis. The purified DNA samples were individually diluted to 1 ng/μL with water. Using the diluted genomic DNA as a template, PCR was performed with the primers 341F: 5′-CCTAYGGGRBGCASCAG-3′ and 806R: 5′-GGACTACNNGGGTATCTAAT-3′ to amplify a specific region of the 16S rDNA gene. The PCR system contained specific primers with barcodes, Phusion^®^ High-Fidelity PCR Master Mixture with GC Buffer, and high-efficiency high-fidelity polymerase from New England Biolabs. PCR products were checked on 2% agarose gel by electrophoresis. According to the concentrations of PCR products, PCR products of the same amount for all the samples were mixed. The target bands were recovered for library construction using a gel recovery kit (Truseq ^®^ DNA PCR-Free Sample Preparation Kit) provided by Qiagen. The constructed library was quantified by Qubit. When the qualified library was constructed, NovaseQ 6000 was then used for sequencing analysis.

### 2.5. Data Analysis

SPSS 22.0 statistical software was used for statistical analysis. One-way analysis of variation (ANOVA) with Duncan’s multiple comparisons was performed for significance test. The differences in alpha diversity index between the groups were determined using the Tukey test and Wilcox test of the Agricolae package. The Vegan package of the R Software was used for NMDS (non-metric multidimensional scaling) analysis. The differences in beta diversity index across samples were also analyzed using the R Software. *p* < 0.05 was set as criterion for significant difference.

## 3. Results

### 3.1. Effects of Dietary Probiotics on Production Performance of Geese

Compared with the control group (the group A), from day 1 to day 70, the ADG of the experimental groups were significantly increased (*p* < 0.05) (Table 2); the ADFI in groups C and D were significantly increased (*p* < 0.05), while there were no significant differences in the feed to gain ratio (F/G) among the groups (*p* > 0.05). These findings indicated that dietary probiotics improved the production performance of geese.

### 3.2. Effects of Dietary Probiotics on Intestinal Growth Index of Geese

Compared with the control group, the absolute weight of duodenum was significantly increased in the treatment groups (*p* < 0.05), but there was no significant difference in the absolute weight of duodenum among the treatment groups (*p* > 0.05) (Table 3). Moreover, there were no significant differences in the absolute weights of jejunum, ileum, and cecum, as well as the absolute lengths of all different intestinal segments (duodenum, jejunum, ileum, and cecum) among all the groups (*p* > 0.05). The relative weights and lengths of all different intestinal segments were also calculated, which showed that the relative lengths but not the weights of all different intestinal segments in all treatment groups were significantly smaller than in control group A because of the increased body weight (*p* < 0.05) (Table S2). These findings indicated that dietary probiotics promoted duodenal growth of geese.

### 3.3. Effects of Dietary Probiotics on Intestinal Antioxidative Capacity

**Figure 1:**
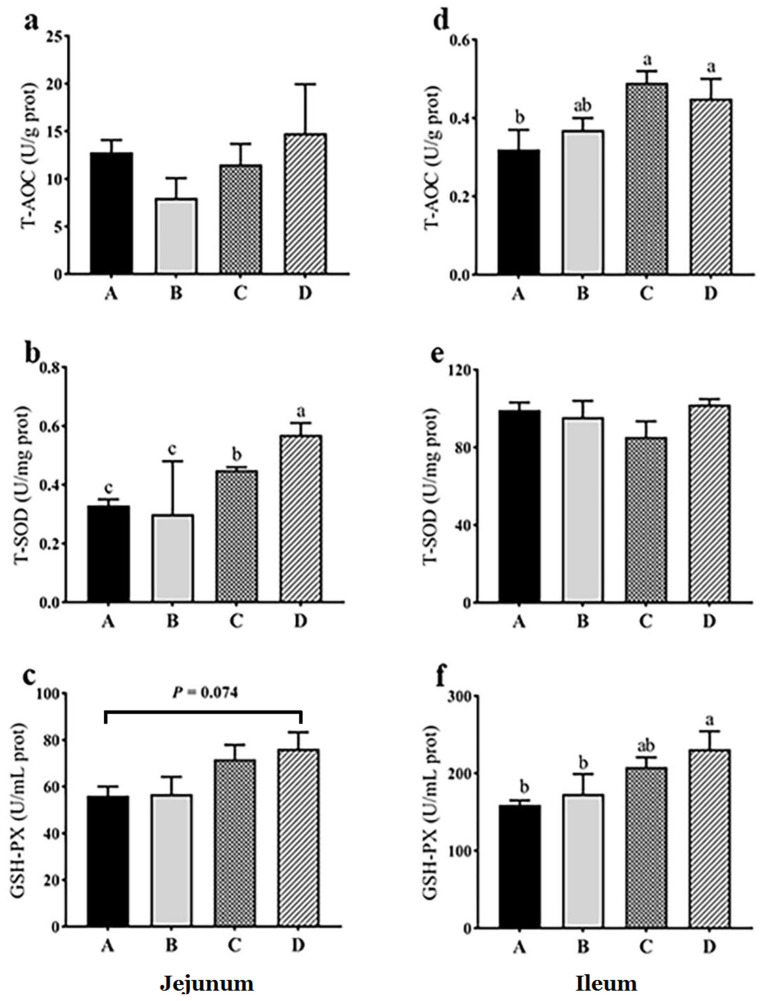
Effects of dietary probiotics on jejunal (**a**–**c**) and ileal (**d**–**f**) antioxidant capacities of geese at the age of 70 days. Note: a and d for total antioxidative capacity (T-AOC), b and e for total activity of superoxide dismutase (T-SOD), c and f for the activity of glutathione peroxidase (GSH-PX). Group A (as control) was fed a basal diet, the treatment groups (B, C and D) were fed the basal diet supplemented with 250 mg/kg *Clostridium butyricum* (viable count 3.0 × 10^6^ CFU/g), 250 mg/kg *Bacillus subtilis* (viable count 2.0 × 10^7^ CFU/g), or the combination of 250 mg/kg *Clostridium butyricum* plus 250 mg/kg *Bacillus subtilis*, respectively. *n* = 6. Different letters above the bars indicate significant differences between the groups (*p* < 0.05). The data are presented as the mean ± SEM.

In jejunal mucosa, there was no significant difference in T-AOC among all groups (*p* > 0.05), while in ileal mucosa, T-AOC was significantly increased in groups C and D compared with group A (*p* < 0.05) (Figure 1). In jejunal mucosa, T-SOD in groups C and D was significantly greater than that in groups A and B, and T-SOD in group D was significantly greater than that in group C (*p* < 0.05), while in ileal mucosa, there were no significant differences in T-SOD among all groups (*p* > 0.05) (Figure 1). In jejunal mucosa, GSH-PX in group D showed an increasing trend compared with group A (*p* = 0.074), and in ileal mucosa, GSH-PX in group D was significantly greater than that in groups A and B (*p* < 0.05) (Figure 1). The results indicated that dietary probiotics generally increased antioxidative capacity in intestinal mucosa.

### 3.4. Effects of Dietary Probiotics on Intestinal Histomorphology

**Figure 2:**
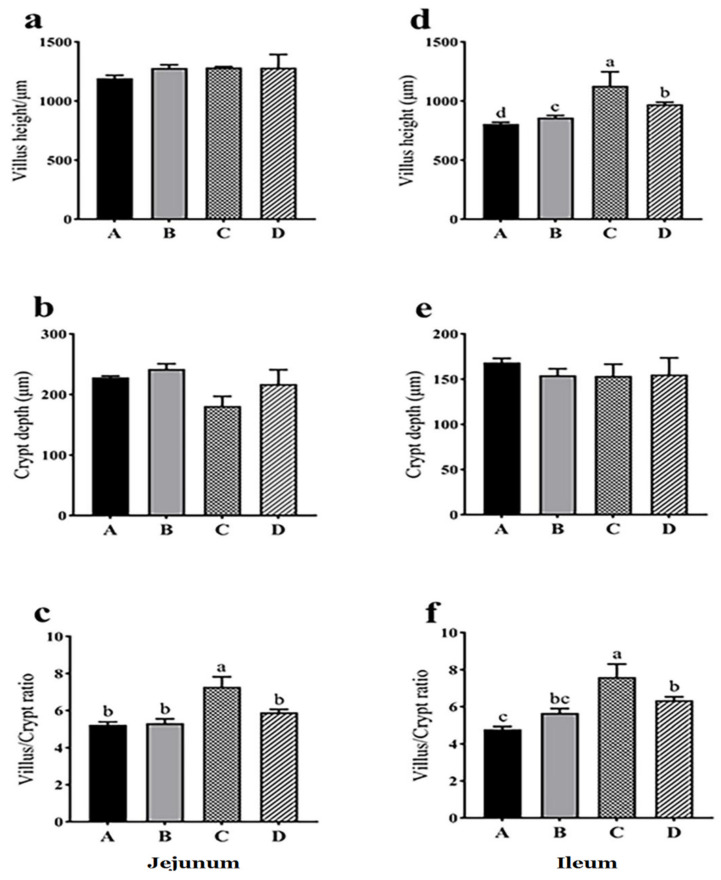
Effects of dietary probiotics on jejunal (**a**–**c**) and ileal (**d**–**f**) histomorphology of geese at the age of 70 days. Note: a and d for villus height, b and e for crypt depth, c and f for the ratio of villus height to crypt depth. Group A (as control) was fed a basal diet, treatment groups (B, C and D) were fed the basal diet supplemented with 250 mg/kg *Clostridium butyricum* (viable count 3.0 × 10^6^ CFU/g), 250 mg/kg *Bacillus subtilis* (viable count 2.0 × 10^7^ CFU/g), or the combination of 250 mg/kg *Clostridium butyricum* plus 250 mg/kg *Bacillus subtilis*, respectively. *n* = 6. Different letters above the bars indicate significant differences between the groups (*p* < 0.05). The data are presented as the mean ± SEM.

The villus height of jejunum was not significantly different among the groups (*p* > 0.05), but that of ileum was significantly different among the groups with the smallest in control group A and the greatest in treatment group C (*p* < 0.05) (Figure 2, Figure S2). The crypt depth in group C showed a decreasing trend compared with group A (*p* = 0.058), and the ratio of villus height to crypt depth in group C was significantly greater than that in groups A, B and D (*p* < 0.05) (Figure 2, Figure S2). In ileum, the ratio of villus height to crypt depth in groups C and D was significantly greater than that in group A (*p* < 0.05), and the ratio in group C was significantly greater than that in group B (*p* < 0.05) (Figure 2, Figure S2). The results indicated that dietary probiotics generally improved intestinal morphological structure.

### 3.5. Effects of Dietary Probiotics on the mRNA Expression of Inflammatory and Tight Junction Related Genes in Intestinal Tissues

**Figure 3:**
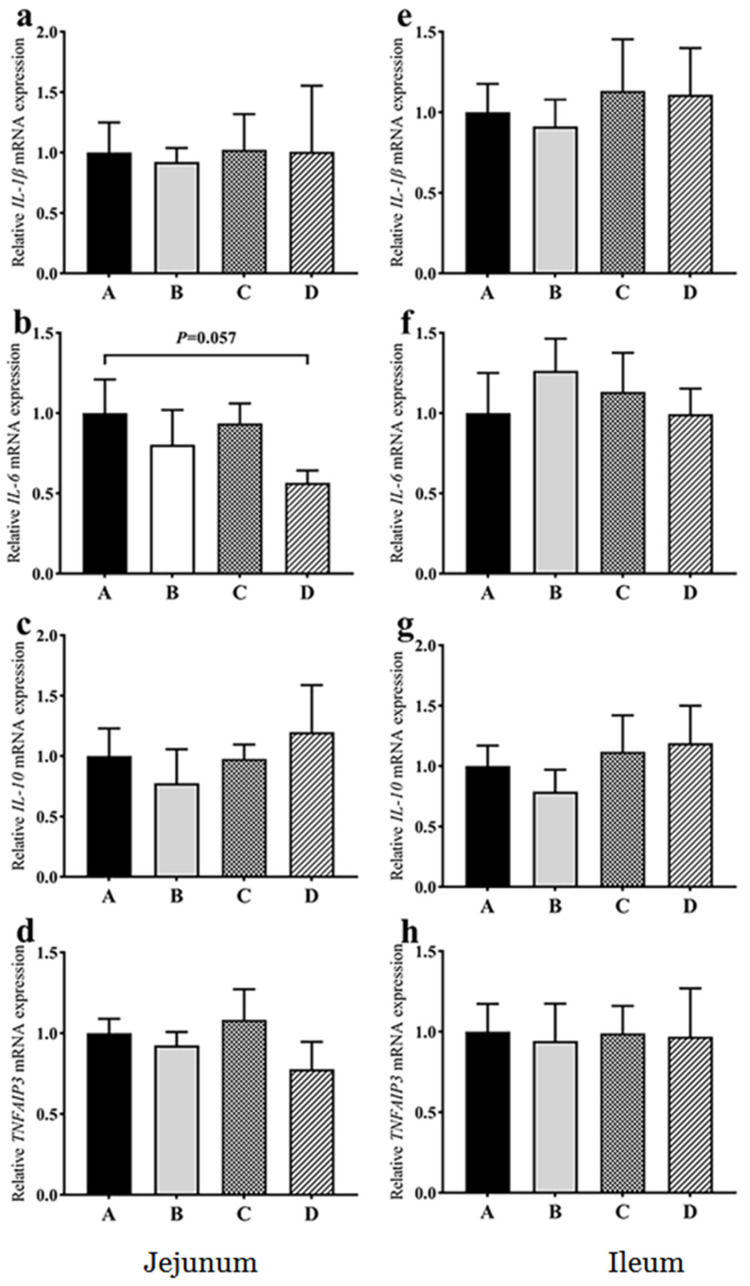
Effects of dietary probiotics on the mRNA expression of inflammatory cytokines in the jejunum (**a**–**d**) and ileum (**e**–**h**) of geese at the age of 70 days. Note: a and e for *IL-1β* gene; b and f for *IL-6* gene; c and g for *IL-10* gene; d and h for *TNFAIP3* gene. Group A (as control) was fed a basal diet, the treatment groups (B, C and D) were fed the basal diet supplemented with 250 mg/kg *Clostridium butyricum* (viable count 3.0 × 10^6^ CFU/g), 250 mg/kg *Bacillus subtilis* (viable count 2.0 × 10^7^ CFU/g), or the combination of 250 mg/kg *Clostridium butyricum* plus 250 mg/kg *Bacillus subtilis*, respectively. The relative mRNA transcript abundance of each gene was determined by quantitative PCR and presented as fold change over control group A. The internal control gene is *GAPDH*. *n* = 6. The data are presented as the mean ± SEM.

**Figure 4:**
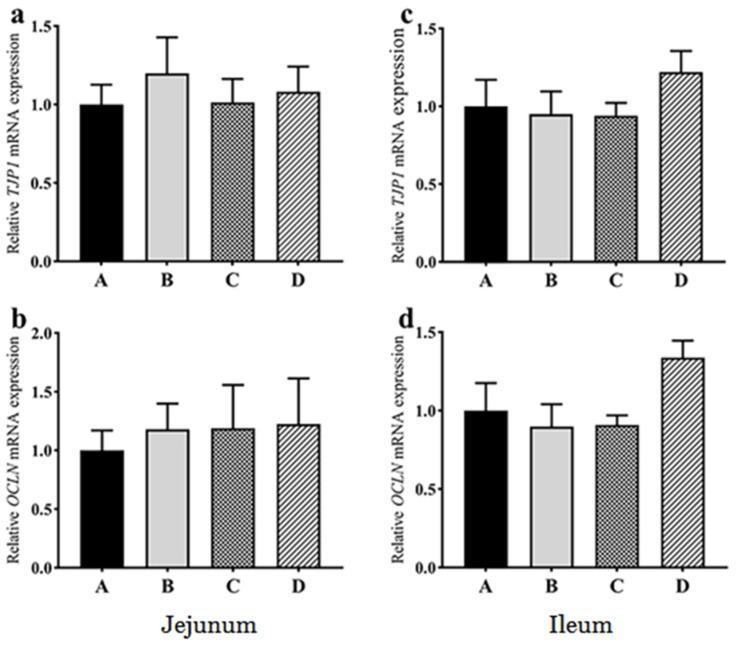
Effects of dietary probiotics on the mRNA expression levels of the tight junction-related genes in the jejunum (**a**,**b**) and ileum (**c**,**d**) of the geese at the age of 70 days. Note: a and c for *TJP1* gene, b and d for *OCLN* gene. Group A (as control) was fed a basal diet, the treatment groups (B, C and D) were fed the basal diet supplemented with 250 mg/kg *Clostridium butyricum* (viable count 3.0 × 10^6^ CFU/g), 250 mg/kg *Bacillus subtilis* (viable count 2.0 × 10^7^ CFU/g), or the combination of 250 mg/kg *Clostridium butyricum* plus 250 mg/kg *Bacillus subtilis*, respectively. The relative mRNA transcript abundance of each gene was determined by quantitative PCR and presented as fold change over control group A. The internal control gene is *GAPDH*. *n* = 6. The data are presented as the mean ± SEM.

Compared with group A, the mRNA expression of *IL-6* in the jejunum showed a decreasing trend in group D (*p* = 0.057) (Figure 3). There were no significant differences in the mRNA expression of *IL-1β*, *IL-10*, and *TNFAIP3* in the jejunum among the groups (Figure 3). There were no significant differences in the mRNA expression of *IL-1β*, *IL-6*, *IL-10* and *TNFAIP3* in the ileum among the groups (Figure 3). Compared with group A, there were no significant differences in the mRNA expression of *TJP1* and *OCLN* genes in the jejunum and ileum of all the treatment groups (Figure 4). These findings indicated that dietary probiotics did not cause inflammation and the change of tight junction in goose intestine.

### 3.6. Effects of Dietary Probiotics on the Alpha Diversity of Intestinal Microflora

The alpha diversity analysis index (observed species, Shannon, Simpson, Chao1, ACE) of different samples was calculated on the threshold of 97% consistency (Table 4). The alpha diversity index of jejunal microflora, such as observed species, Chao1 and ACE, in group A were significantly greater than those in all the treatment groups (*p* < 0.01). Moreover, there were no significant differences in the alpha diversity index among the treatment groups. In addition, NMDS-based Bray-Curtis distance analysis with the taxa information of intestinal microflora across different samples indicated that the taxa distribution of jejunal microflora in group A was far from that in the treatment groups; whereas the taxa distributions of jejunal microflora were close to each other among all the treatment groups (Figure S1). This finding is in line with the finding that dietary probiotics supplementation dramatically change the composition of jejunal microflora, which is indicated by the alpha diversity analysis. In contrast, there was no significant difference in the alpha diversity of ileal microflora among all the groups, and the taxa distribution of ileal microflora in group A was not far from those of groups B and C, except group D (Figure S1). The results together indicated that dietary probiotics reduced the diversity of microflora in jejunum but not ileum.

### 3.7. Effects of Dietary Probiotics on the Composition of Intestinal Microflora

**Figure 5:**
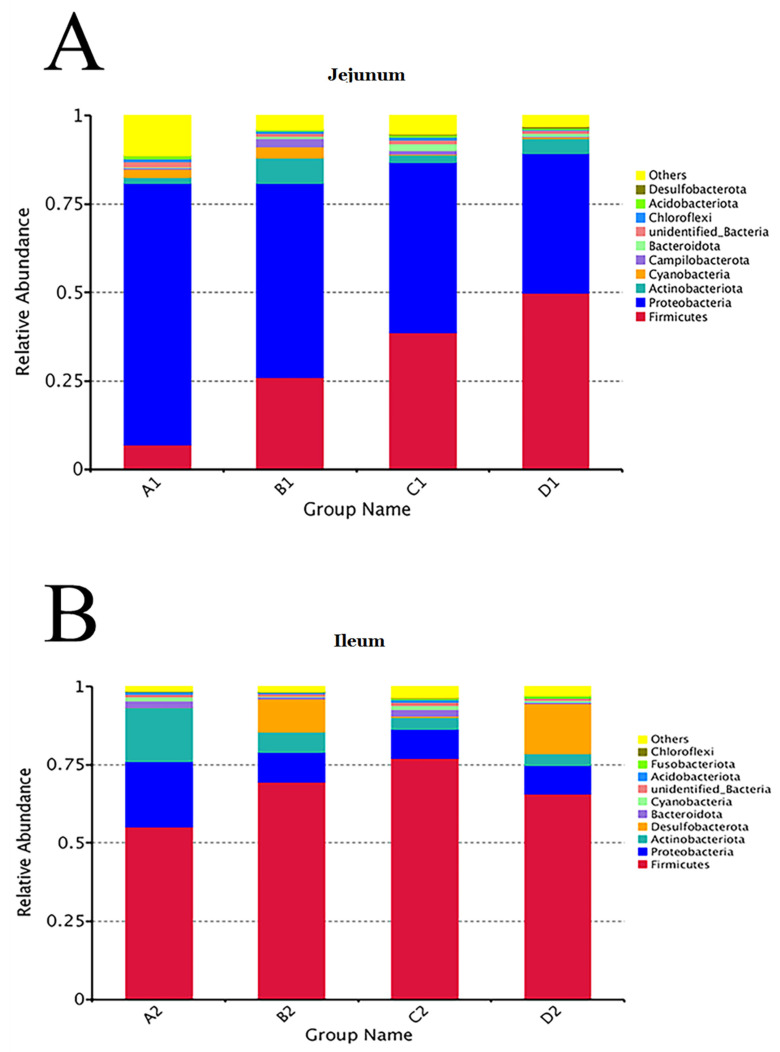
Relative abundance of bacteria in jejunal (**A**) and ileal (**B**) contents at phylum level. Note: Group A (as control) was fed a basal diet, the treatment groups (B, C and D) were fed the basal diet supplemented with 250 mg/kg *Clostridium butyricum* (viable count 3.0 × 10^6^ CFU/g), 250 mg/kg *Bacillus subtilis* (viable count 2.0 × 10^7^ CFU/g), or the combination of 250 mg/kg *Clostridium butyricum* plus 250 mg/kg *Bacillus subtilis*, respectively. *n* = 6.

**Figure 6:**
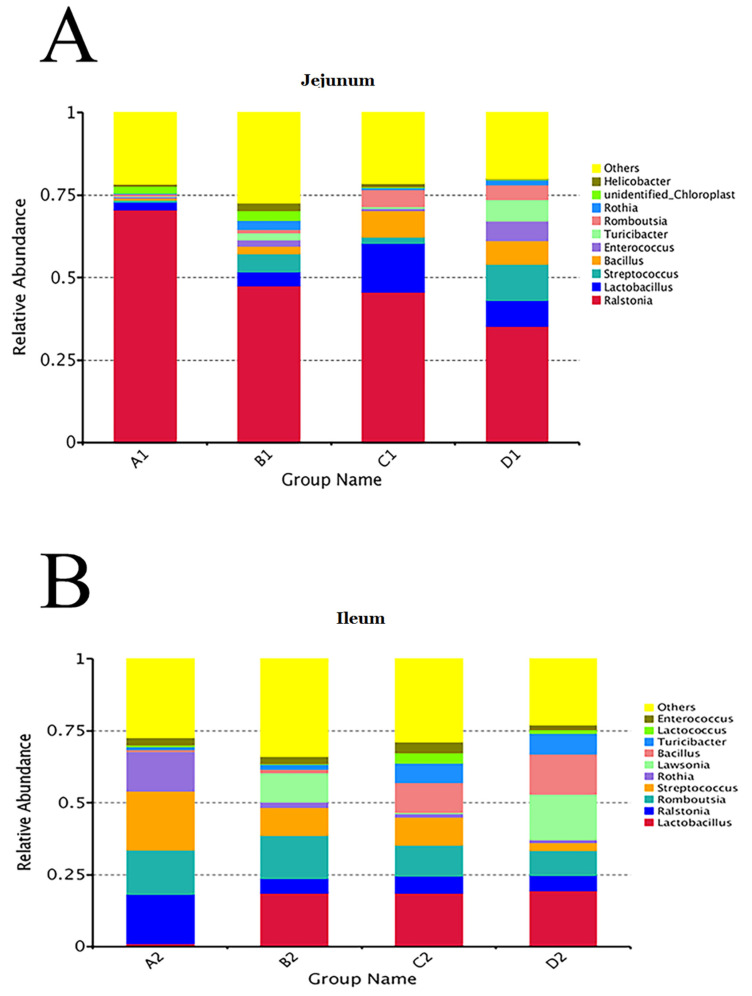
Relative abundance of bacteria in jejunal (**A**) and ileal (**B**) contents at genus level. Note: Group A (as control) was fed a basal diet, the treatment groups (B, C and D) were fed the basal diet supplemented with 250 mg/kg Clostridium butyricum (viable count 3.0 × 10^6^ CFU/g), 250 mg/kg Bacillus subtilis (viable count 2.0 × 10^7^ CFU/g), or the combination of 250 mg/kg Clostridium butyricum plus 250 mg/kg Bacillus subtilis, respectively. *n* = 6.

The top 10 taxa of jejunal and ileal microflora were shown at the phylum and genus levels in Figure 5 and Figure 6. At the phylum level, the relative abundances of *Proteobacteria* in the jejunal contents of groups A, B, C, and D were 73.99%, 54.90%, 48.09%, and 39.45%%, respectively. The relative abundances of *Firmicutes* in the jejunal contents of groups A, B, C, and D were 6.93%, 25.94%, 38.74%, and 49.88%, respectively. Moreover, in the ileal contents of groups A, B, C, and D, the relative abundance of *Firmicutes* was the greatest, which was 55.21%, 69.50%, 77.08%, and 65.70%, respectively. At the genus level, the relative abundances of *Ralstonia* in the jejunal contents of groups A, B, C, and D were 70.50%, 47.60%, 45.70%, and 45.70%, respectively. As the second abundant genus, the relative abundances of *Lactobacillus* in group A, *Streptococcus* in group B, *Lactobacillus* in group C, and *Streptococcus* in group D were 2.39%, 5.50%, 14.78%, and 10.99%, respectively. In the ileum, the genera of bacteria with the greatest relative abundance were *Streptococcus* (20.56%) in group A, *Lactobacillus* (18.69%) in group B, *Lactobacillus* (18.59%) in group C, and *Lactobacillus* (19.43%) in group D. The results indicated that dietary probiotics decreased the relative abundance of the dominant *Proteobacteria* phylum and the dominant *Ralstonia* genus and increased the relative abundances of the dominant *Firmicutes* phylum and the dominant *Lactobacillus* genus in intestinal microflora.

### 3.8. The Effects of Dietary Probiotics on the Functions of Intestinal Bacteria

**Figure 7:**
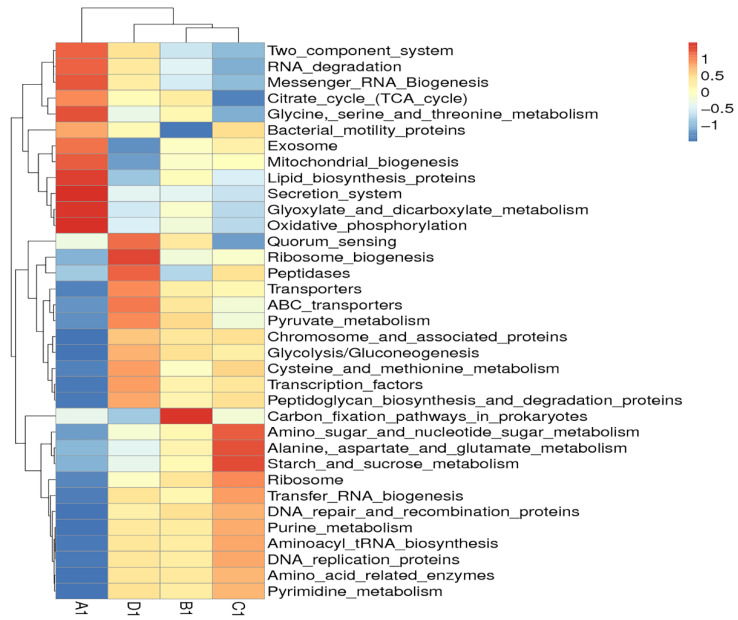
Hierarchical clustering heatmap of Tax4fun annotated function of bacteria in the jejunal content. Note: The heatmap was constructed at the KEGG level 3 using the relative abundance of bacteria in the jejunal contents from different groups of geese. The red or blue colors for a certain annotated function indicate that the relative abundance of bacteria in this group is greater or lower than the average relative abundance of bacteria across all the groups after normalization, respectively. Group A (as control) was fed a basal diet, the treatment groups (B, C and D) were fed the basal diet supplemented with 250 mg/kg *Clostridium butyricum* (viable count 3.0 × 10^6^ CFU/g), 250 mg/kg *Bacillus subtilis* (viable count 2.0 × 10^7^ CFU/g), or the combination of 250 mg/kg *Clostridium butyricum* plus 250 mg/kg *Bacillus subtilis*, respectively. *n* = 6.

**Figure 8:**
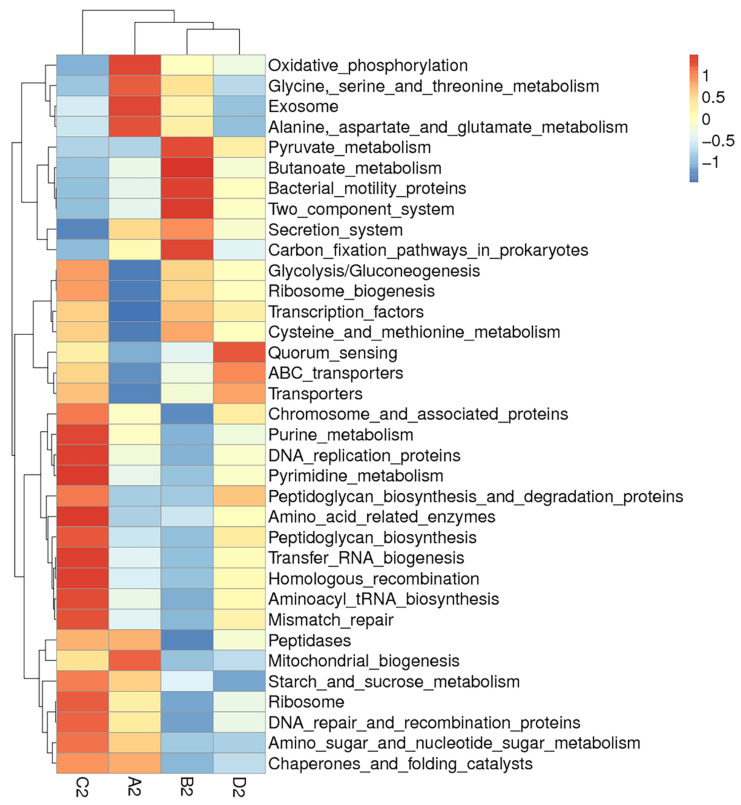
Hierarchical clustering heatmap of Tax4fun annotated function of bacteria in the ileal content. Note: The heatmap was constructed at the KEGG level 3 using the relative abundance of bacteria in the ileal contents from different groups of geese. The red or blue colors for a certain annotated function indicate that the relative abundance of bacteria in this group is greater or lower than the average relative abundance of bacteria across all the groups after normalization, respectively. Group A (as contro)l was fed a basal diet, the treatment groups (B, C and D) were fed the basal diet supplemented with 250 mg/kg Clostridium butyricum (viable count 3.0 × 10^6^ CFU/g), 250 mg/kg Bacillus subtilis (viable count 2.0 × 10^7^ CFU/g), or the combination of 250 mg/kg Clostridium butyricum plus 250 mg/kg Bacillus subtilis, respectively. *n* = 6.

Clustering analysis of bacteria function showed that the predicted functions of jejunal or ileal microflora in group A were quite different from those of all the treatment groups (Figure 7 and Figure 8). In jejunum, the predicted functions of microflora including the secretion system and oxidative phosphorylation in group A were much stronger than those of all the treatment groups. In ileum, the predicted functions of microflora including oxidative phosphorylation, exosome, and mitochondrial biogenesis in group A were much stronger than those of all the treatment groups. Moreover, each treatment group had its own unique bacterial functions enhanced by dietary probiotics. In jejunum, group B was characterized by functions such as carbon fixation pathways in prokaryotes; group C was characterized by functions such as starch and sucrose metabolism, alanine, aspartate, and glutamate metabolism; and group D was characterized by functions such as ribosome biogenesis and peptidases. In ileum, group B was characterized by functions such as butanoate metabolism and two component system; group C was characterized by functions such as purine metabolism and pyrimidine metabolism; and group D was characterized by functions such as quorum sensing and transporters. The results indicated that dietary probiotics significantly altered the functions of intestinal microflora.

## 4. Discussion

Gut microbiota can help digest food, produce bioactive substances, constitute intestinal biological barrier, and maintain intestinal health, etc. Thus, it is key to improving the production performance and health of animals. Probiotics, including beneficial bacteria, have been widely used in animal production as probiotics can improve the composition of gut microbiota and production performance. In this study, we demonstrated that supplementing diet with probiotics (*Clostridium butyricum*, *Bacillus subtilis* or their combination) enhanced production performance, promoted duodenal growth, increased antioxidative capacity in intestinal mucosa, improved intestinal morphological structure, reduced the diversity of microflora in jejunum, modulated the composition of intestinal microflora, and altered intestinal functions without causing inflammation and changing intestinal tight junction in geese, which has rarely been reported previously.

The finding that dietary probiotics promoted goose growth (ADG) and food intake (ADFI) in this study is consistent with previous broiler studies [8,9]. *Clostridium butyricum* can produce butyric acid and other short-chain fatty acids, which can regulate food intake and energy metabolism, maintain the stability of intestinal epithelial cells, enhance nutrient absorption and utilization, and increase body weight. The metabolites produced by *Clostridium butyricum* can also reduce the environmental pH and prevent the proliferation of harmful bacteria [10]. Moreover, *Bacillus subtilis* as beneficial bacteria in animal intestine can synthesize amino acids, vitamins, and other substances, which have a positive effect on animal production performance [11]. *Bacillus subtilis* can also produce a large number of digestive enzymes that promote food digestion and mitigate the effect of anti-nutritional factors, thus facilitating nutritional absorption and utilization [12,13]. In addition, *Bacillus subtilis* can produce antimicrobial peptides and other substances to inhibit the proliferation of harmful bacteria in the intestinal track.

Probiotic bacteria do not only exert the direct effects mentioned above, but also indirectly affect animal production performance by modulating the compositions of intestinal microflora and their metabolites. Indeed, in this study, dietary probiotics decreased the relative abundances of the dominant *Proteobacteria* phylum and the dominant *Ralstonia* genus, and increased the relative abundances of the dominant *Firmicutes* phylum and the dominant *Lactobacillus* genus. Previous studies indicate that some bacteria in *Proteobacteria* phylum or *Ralstonia* genus are harmful to animal health, and thus the increase in the abundance of bacteria belonging to *Proteobacteria* phylum or *Ralstonia* genus may reflect the elevated health risk and the increased instability of intestinal microflora [14,15]. In contrast, the increase in the abundance of bacteria belonging to *Firmicutes* phylum or *Lactobacillus* genus is generally beneficial to host animals. Previous studies indicate that the major function of *Firmicutes* phylum is the hydrolysis of protein and carbohydrate [16], and the abundance of *Firmicutes* is positively correlated with animal growth performance [17]. Moreover, *Lactobacilli*, the bacteria that are more tolerant to and better proliferate in acidic environments, can generate a large amount of lactic acid, thus improving the intestinal environment and preventing the proliferation of harmful bacteria [18]. Consistently, in this study, clustering analysis on bacterial function at KEGG level 3 showed dietary probiotics altered intestinal bacterial functions, such as the enhanced peptidases, carbon fixation, and metabolic function of starch and sugar. In vivo and in vitro studies have shown that bioactive peptides have a large number of biological functions, such as immune regulation, antimicrobial activity, and antioxidation [19]. For example, some studies indicate that biopeptides have beneficial effects against oxidative stress and inflammatory responses [20]. In line with this, dietary probiotics increased the antioxidative capacity of intestinal mucosa without the induction of proinflammatory cytokines in goose intestine. Taken together, the findings from this study suggest that probiotic bacteria promote animal growth and maintain intestinal health by modulating the composition and function of intestinal microflora.

In this study, dietary probiotics promoted duodenal growth and increased the ratio of villus height to crypt depth, which may also contribute to the increase of goose body weight. Previous studies indicate that butyric acid, the metabolite of *Clostridium butyricum*, plays an important role in the regeneration of intestinal epithelial tissue. Supplementing *Clostridium butyricum* into the diet can improve intestinal morphology of broilers [21,22]. On the other hand, the supplementation of 1 × 10^6^ CFU/kg *Bacillus subtilis* into the diet can increase villus height of jejunum in broilers [23]. In addition to the growth promoting effects of bioactive metabolites such as butyric acid produced by probiotic bacteria, the bacteria may prevent damage to intestinal epithelia by inhibiting proliferation of harmful bacteria in the intestine. The intestine is the organ responsible for the digestion of food and absorption of nutrition, the increases of intestinal weight and the ratio of villus height to crypt depth indicate that the function of the intestine is enhanced [24]. The results of this study therefore suggest that dietary probiotics promote the growth of geese by enhancing intestinal function.

In this study, dietary probiotics did not induce the expression of pro/inflammatory cytokines such as Tumor Necrosis Factor-α (*TNF-α*), *IL-1β*, *IL-6* and *IL-10*, which is consistent with previous findings showing the anti-inflammation effect of probiotics. Liu et al. found that in the model of corticosterone-induced liver injury, supplementing 400 mg/kg *Clostridium butyricum* to the diet could inhibit the expression of genes related to the inflammatory response and promote the expression of genes related to anti-inflammatory response in Beijing ducks [25]. Moreover, lipopolysaccharide (LPS) injections into piglets fed a diet supplemented with *Clostridium butyricum* for 36 days did not induce the expression of *TNF-α* [26]. Similarly, *Bacillus subtilis* could effectively improve the immunity of livestock and poultry, enhance the expression of anti-inflammatory genes, and stimulate the growth and development of immune organs [9,27,28]. LPS is a key trigger of inflammation and immune response, the increased proliferation of harmful bacteria in the intestine can induce inflammation, immune response, and damage to the intestine via LPS, thus, probiotics supplementation may prevent intestinal disorders, damage, and unnecessary activation of the immune system by inhibiting proliferation of harmful bacteria [6]. In addition, the anti-inflammatory effects of probiotics may be partially attributed to the role of butyric acid produced by *Clostridium butyricum* in the repair of intestinal epithelial tissue and the intestinal barrier formed by probiotic bacteria, preventing the damage to intestinal tissue caused by harmful intestinal bacteria [29].

Interestingly, this study indicated that dietary probiotics increased the antioxidative capacity of intestinal mucosa, which may be a reason for no occurrences of inflammation in the intestines of geese fed a diet supplemented with probiotics. Previous studies demonstrated that *Clostridium butyricum* could remove reactive oxygen species in vivo by generating reductive coenzyme Ⅱ peroxidase [30]. Indeed, *Clostridium butyricum* can relieve oxidative stress induced by CCL4 in mice [31]. The mechanism may be that *Clostridium butyricum* can produce some digestive enzymes, butyric acid, and hydrogen, thus affecting the activity of antioxidative enzymes [32]. For *Bacillus subtilis*, Wang et al. reported that serum T-SOD and GSH-PX activities of broiler chickens fed a diet supplemented with *Bacillus subtilis* was significantly greater than those of the control chickens fed a diet without supplementation [33]. Liu et al. also found that the supplementation of *Bacillus subtilis* to the diet could significantly increase the serum GSH-PX content of laying breeders [34]. The mechanism underlying the induction of antioxidative capacity by probiotics, however, warrants further investigation.

It is noteworthy that dietary probiotics reduced the α diversity of intestinal microflora in this study, which seems to be contradictory to the notion that diseases are usually associated with decreased diversity of gut microbiota, and vice versa. For example, Li et al. reported that a reduced α diversity of gut microbiota was seen in the hens with fatty livers vs. normal livers [35]. In broilers, *Bacillus subtilis* supplementation to the diet for 21 days significantly increased microbial diversity in the jejunum [36]. As the dosage of supplemented probiotics and the intestinal physiology could affect the composition of intestinal microflora, it is worth testing whether the differences in dosage of probiotics or the intestinal physiology among different studies account for this contradiction. Moreover, this study showed that dietary probiotics had stronger effects in upper intestinal tracts (duodenum and jejunum) than lower intestinal tracks (ileum and cecum), which is indicated by intestinal weight and microbial diversity. This is not difficult to explain as supplemented probiotic always move from upper to lower intestinal tracks. Additionally, previous studies indicate that *Clostridium butyricum* can promote tight junction-related genes such as *OCLN* and *TJP1* via its metabolite butyric acid, this study, however, did not show this effect. The reason for this contradiction needs to be further investigated.

## 5. Conclusions

In conclusion, dietary probiotics promote the growth of geese, which could be attributed to their positive effects on intestinal structure and function, the composition and functions of gut microflora, and intestinal antioxidative capacity, without causing inflammation and changes of tight junction in the intestine.

## Figures and Tables

**Table 1:** The information on the primers used in quantitative PCR.

Gene	Primer Sequence (5′–3′)	Genbank Accession Number
IL-6	F: AACCTCAACCTCCCCAA	XM_013171777. 1
	R:CAGCGACTCAACTTTTT	
IL-10	F: CATCAAGAACAGCGAGC	XM_013189578. 1
	R: CATCCTTTTCAAACGTC	
IL-1β	F: CCGCTTCATCTTCTACCG	XM_013199176. 1
	R: TGTAGGTGGCGATGTTGAC	
TNFAIP3	F:GAATGAACCCTCCTCCG	XM_013175522. 1
	R:ATCTGGTAACAGGAAGG	
TJP1	F:ACGCTGTTGAATGTCCC	XM_013177396. 1
	R:TCGAAGACTGCCGTTGC	
OCLN	F:GGAGCAGCCCAGGAAAG	XM_013199669. 1
	R:GCTTGAGGTCGGTGTCG	
GAPDH	F:GCCATCAATGATCCCTTCAT	XM_013199522. 1
	R:CTGGGGTCACGCTCCTG	

**Table 2:** Effects of probiotics supplementation on goose production performance.

Group		A	B	C	D	*p* Value
1 d	Body Weight (BW)	95.28 ± 3.40	95.28 ± 1.95	91.94 ± 4.40	93.33 ± 1.82	0.206
28 d	BW	1450.18 ± 100.00^b^	1472.88 ± 76.71^a^	1567.33 ± 32.02^a^	1532.16 ± 115.73^a^	0.011
70 d	BW	3369.88 ± 198.21^b^	3705.15 ± 201.35^a^	3853.92 ± 196.43^a^	3781.33 ± 367.51^a^	0.017
1–28 d	ADG	48.39 ± 3.64	49.20 ± 3.73	52.69 ± 1.14	51.39 ± 4.15	0.099
	ADFI	109.48 ± 6.31	110.43 ± 5.69	115.80 ± 5.23	113.47 ± 7.80	0.318
	F/G	2.27 ± 0.15	2.25 ± 0.19	2.20 ± 0.10	2.22 ± 0.21	0.883
29–70 d	ADG	45.71 ± 2.88^b^	53.15 ± 5.75^a^	54.44 ± 5.14^a^	53.55 ± 7.16^a^	0.042
	ADFI	246.74 ± 10.68^c^	262.16 ± 7.99^b^	278.81 ± 14.29^a^	271.67 ± 15.55^ab^	0.002
	F/G	5.41 ± 0.24	4.97 ± 0.40	5.17 ± 0.69	5.15 ± 0.81	0.633
1–70 d	ADG	46.78 ± 2.85^b^	51.57 ± 2.87^a^	53.74 ± 2.82^a^	52.69 ± 5.26^a^	0.016
	ADFI	191.84 ± 8.44^c^	201.46 ± 5.82^bc^	213.61 ± 8.26^a^	208.39 ± 11.18^ab^	0.002
	F/G	4.11 ± 0.18	3.91 ± 0.18	3.99 ± 0.31	3.99 ± 0.52	0.784

Note: Group A (as control) was fed a basal diet, the treatment groups (B, C and D) were fed the basal diet supplemented with 250 mg/kg *Clostridium butyricum* (viable count 3.0 × 10^6^ CFU/g), 250 mg/kg *Bacillus subtilis* (viable count 2.0 × 10^7^ CFU/g), or the combination of 250 mg/kg *Clostridium butyricum* plus 250 mg/kg *Bacillus subtilis*, respectively. The different superscripts in the same row denote the means are significantly different between the groups (*p* < 0.05). ADG denotes the average daily gain, ADFI denotes the average daily feed intake, F/G denotes the feed to gain ratio. The data are presented as the mean ± SEM. *n* = 6.

**Table 3:** Effects of probiotics supplementation on the absolute lengths and weights of different intestinal segments in the geese at the age of 70 days.

	Group	A	B	C	D	*p* Value
Length (cm)	duodenum	34.5 ± 3.53	36 ± 3.14	36.67 ± 1.24	35.54 ± 2.71	0.399
	jejunum	83.88 ± 2.67	83.88 ± 4.72	83.58 ± 3.70	86.58 ± 2.97	0.450
	ileum	77.21 ± 3.83	77.13 ± 3.65	77.46 ± 2.60	80.58 ± 4.52	0.333
	cecum	41.33 ± 3.65	40.38 ± 2.54	41.38 ± 4.54	42.04 ± 2.96	0.875
Weight (g)	duodenum	12.04 ± 1.30^b^	14.67 ± 1.91^a^	15.38 ± 1.36^a^	15.46 ± 2.08^a^	0.007
	jejunum	28.83 ± 4.38	33.79 ± 2.93	33.88 ± 5.27	33.25 ± 2.73	0.676
	ileum	27.29 ± 5.30	29.96 ± 3.31	29.79 ± 4.82	28.04 ±2.86	0.631
	cecum	6.71 ± 1.22	8.08 ± 1.19	7.42 ± 1.28	6.92 ± 0.52	0.118
W/L (g/cm)	duodenum	0.35 ± 0.02	0.41 ± 0.01	0.42 ± 0.01	0.44 ± 0.04	0.051
	jejunum	0.34 ± 0.02^b^	0.40 ± 0.01^a^	0.40 ± 0.02^a^	0.38 ± 0.01^b^	0.047
	ileum	0.35 ± 0.02	0.39 ± 0.01	0.38 ± 0.02	0.35 ± 0.01	0.227
	cecum	0.16 ± 0.01^b^	0.20 ± 0.01^a^	0.18 ± 0.01^ab^	0.16 ± 0.01^b^	0.009

Note: Group A (as control) was fed a basal diet, the treatment groups (B, C and D) were fed the basal diet supplemented with 250 mg/kg *Clostridium butyricum* (viable count 3.0 × 10^6^ CFU/g), 250 mg/kg *Bacillus subtilis* (viable count 2.0 × 10^7^ CFU/g), or the combination of 250 mg/kg *Clostridium butyricum* plus 250 mg/kg *Bacillus subtilis*, respectively. The different superscripts in the same row denote the means are significantly different between the groups (*p* < 0.05). The data are presented as the mean ± SEM. *n* = 6. W/L denotes the weight/length ratio of each intestinal segment.

**Table 4:** The alpha diversity analysis of microbiome in the jejunum of the geese at the age of 70 days.

Intestines	Group	Observed Species	Shannon	Simpson	Chao1	ACE
Jejunum	A	2014 ± 212.61^a^	3.53 ± 0.60	0.49 ± 0.07	2803.92 ± 377.12^a^	3099.14 ± 410.91^a^
	B	731 ± 97.47^b^	3.73 ± 0.74	0.66 ± 0.13	1015.09 ± 161.16^b^	1057.89 ± 200.14^b^
	C	847 ± 161.85^b^	3.37 ± 0.49	0.61 ± 0.09	1124.53 ± 149.85^b^	1210.56 ± 170.31^b^
	D	849 ± 186.34^b^	3.85 ± 0.60	0.73 ± 0.09	1147.94 ± 263.51^b^	1247.34 ± 301.05^b^
	*p* value	<0.001	0.944	0.446	<0.001	<0.001
Ileum	A	805.67 ± 109.18	3.90 ± 0.61	0.66 ± 0.09	1184.02 ± 193.79	1255.22 ± 201.18
	B	778.17 ± 100.71	3.89 ± 0.52	0.69 ± 0.09	1089.46 ± 156.84	1170.38 ± 171.21
	C	1134.40 ± 240.61	5.03 ± 0.41	0.87 ± 0.03	1708.25 ± 504.40	1908.66 ± 570.60
	D	891.00 ± 315.99	3.88 ± 0.94	0.68 ± 0.15	1462.91 ± 644.19	1663.77 ± 799.07
	*p* value	0.520	0.484	0.399	0.625	0.581

Note: Group A (as control) was fed a basal diet, the treatment groups (B, C and D) were fed the basal diet supplemented with 250 mg/kg *Clostridium butyricum* (viable count 3.0 × 10^6^ CFU/g), 250 mg/kg *Bacillus subtilis* (viable count 2.0 × 10^7^ CFU/g), or the combination of 250 mg/kg *Clostridium butyricum* plus 250 mg/kg *Bacillus subtilis*, respectively. The different superscripts in the same column denote the means are significantly different between the groups (*p* < 0.05). *n* = 6. The data are presented as the mean ± SEM.

## Data Availability

The data presented in this study are available on request from the corresponding author.

## Supplementary Materials

The following are available online at https://www.mdpi.com/article/10.3390/ani11113174/s1, Figure S1: NMDS analysis on the diversity of jejunal (A) and ileal (B) microflora, Figure S2: The representative images showing the effects of dietary probiotics on jejunal and ileal histomorphology of geese at the age of 70 days, Table S1: The ingredient and nutritional compositions of basal diet, Table S2: Effects of probiotics supplementation on the relative lengths and weights of different intestinal segments in the geese at the age of 70 days.

## Abbreviations

The following abbreviations are used in this manuscript:

GSH-PX	Glutathione peroxidase
T-SOD	Total superoxide dismutase
T-AOC	Total antioxidative capacity
Il-1β	Interleukin-1β
Il-6	Interleukin-6
Il-10	Interleukin-10
TNFAIP3	Tumor necrosis factor alpha induced protein 3
OCLN	Occludin
TJP1	Tight junction protein 1
