# Supplementary material for: Dietary Clostridium butyricum and Bacillus subtilis Promote Goose Growth by Improving Intestinal Structure and Function, Antioxidative Capacity and Microbial Composition
Source: Animals (Basel). 2021 Nov 6;11(11):3174. doi: 10.3390/ani11113174 (PMC8614425; doi:10.3390/ani11113174)
Supplement: Supplementary file 1 [file animals-11-03174-s001.zip › animals-1417192-supplementary.pdf]

Table S1. The ingredient and nutritional compositions of basal diet.

	**Day 1 to day 28**	**Day 29 to day 70**
**Ingredient composition (%)**		
Corn	48.9	47.2
Bean pulp	30.5	15.1
Wheat middling	10.0	13.7
Rice polishing powder	0.0	8.0
Soybean oil	1.5	0
Rice husk	0.0	3.0
$CaHPO_4$	1.5	0.9
Limestone	1.3	1.3
Rice bran	5.0	9.5
Premix[a]	1.0	1.0
NaCl	0.3	0.3
Total	100	100
**Nutritional composition**[b]		
Metabolizable energy (MJ/Kg)	11.88	11.58
Crude protein	19.00	15.00
Crude Fiber	2.92	3.77
Calcium	1.00	0.90
Total phosphorus	0.69	0.68
Methionine	0.40	0.36

Note: [a)] The content of premix per kilogram (day 1 to day 28): VA 1200000 IU, VD_3 400000 IU, VE 1800 IU, VK 150 mg, VB_1 90 mg, VB_2 800 mg, VB_6 320 mg, VB_{12} 1 mg, nicotinic acid 4.5 g, calcium pantothenate 1100 mg, folic acid 65 mg, biotin 5 mg, choline 45 Mg, Fe 6 g, Cu 1 g, Mn 9.5 g, Zn 9 g, I 50 mg, Se 30 mg. The content of premix per kilogram (day 29 to day 70): VA 1200000 IU, VD_3 400000 IU, VE 1800 IU, VK 150 mg, VB_1 60 mg, VB_2 600 mg, VB_6 200 mg, VB_{12} 1 mg, nicotinic acid 3 g, calcium pantothenate 900 mg, folic acid 50 mg, biotin 4 mg, choline 35 mg, Fe 6 g, Cu 1 g, Mn 9.5 g, Zn 9 g, I 50 mg, Se 30 mg. [b)] Metabolic energy was measured in nutrient level, and the rest was calculated.

Table S2. Effects of probiotics supplementation on the relative lengths and weights of different intestinal segments in the geese at the age of 70 days.

group	duodenum		jejunum		ileum		cecum	
	length	weight	length	weight	length	weight	length	weight
A	11.03 ± 1.04[a]	3.79 ± 0.38	26.92 ± 1.73[a]	9.18 ± 1.71	24.80 ± 2.09[a]	8.68 ± 1.99	13.27 ± 1.45[a]	2.12 ± 0.43
B	9.86 ± 0.80[b]	3.99 ± 0.52	23.05 ± 1.09[b]	9.27 ± 0.73	21.19 ± 1.23[b]	8.21 ± 0.94	11.07 ± 0.80[b]	2.22 ± 0.28
C	9.66 ± 0.30[b]	4.04 ± 0.29	22.03 ± 0.59[b]	8.87 ± 1.24	20.433 ± 0.84[b]	7.83 ± 1.17	10.94 ± 0.95[b]	1.96 ± 0.30
D	9.76 ± 0.85[b]	4.20 ± 0.59	23.77 ± 2.16[b]	9.09 ± 1.10	22.21 ± 2.32[b]	7.71 ± 1.13	11.53 ± 0.73[b]	1.89 ± 0.13

Note: The group A as control was fed a basal diet, the treatment groups (B, C and D) were fed the basal diet supplemented with 250 mg/kg *Clostridium butyricum* (viable count was 3.0×10^6 CFU/g), 250 mg/kg *Bacillus subtilis* (viable count was 2.0×10^7 CFU/g), or the combination of 250 mg/kg *Clostridium butyricum* plus 250 mg/kg *Bacillus subtilis*, respectively. The different superscripts in the same row denote the means are significantly different between the groups ($p < 0.05$). The data are presented as the mean ± SEM. $n = 6$.

A

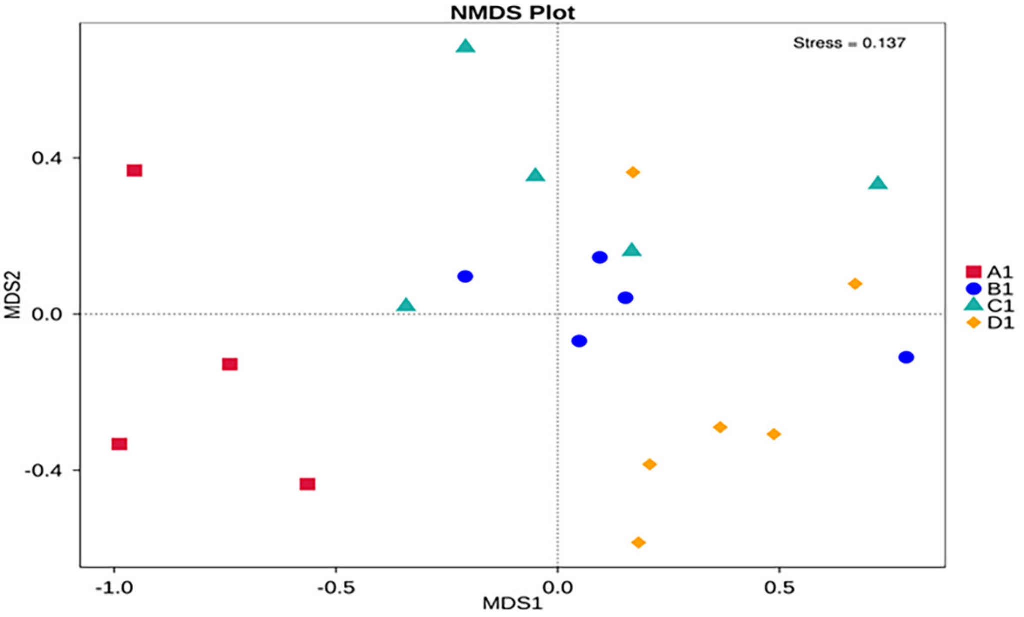

B

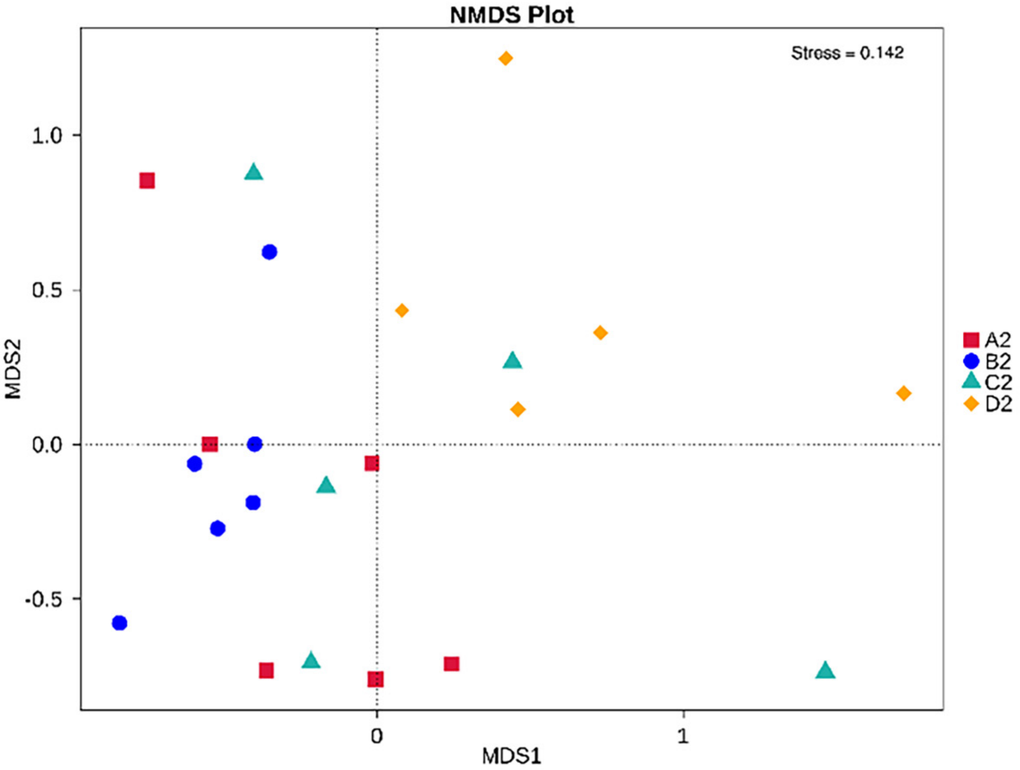

Figure S1. NMDS analysis on the diversity of jejunal (**A**) and ileal (**B**) microflora. Note: The group A as control was fed a basal diet, the treatment groups (B, C and D) were fed the basal diet supplemented with 250 mg/kg Clostridium butyricum (viable count was 3.0×10^6 CFU/g), 250 mg/kg Bacillus subtilis (viable count was 2.0×10^7 CFU/g), or the combination of 250 mg/kg *Clostridium butyricum* plus 250 mg/kg *Bacillus subtilis*, respectively. $n = 6$.

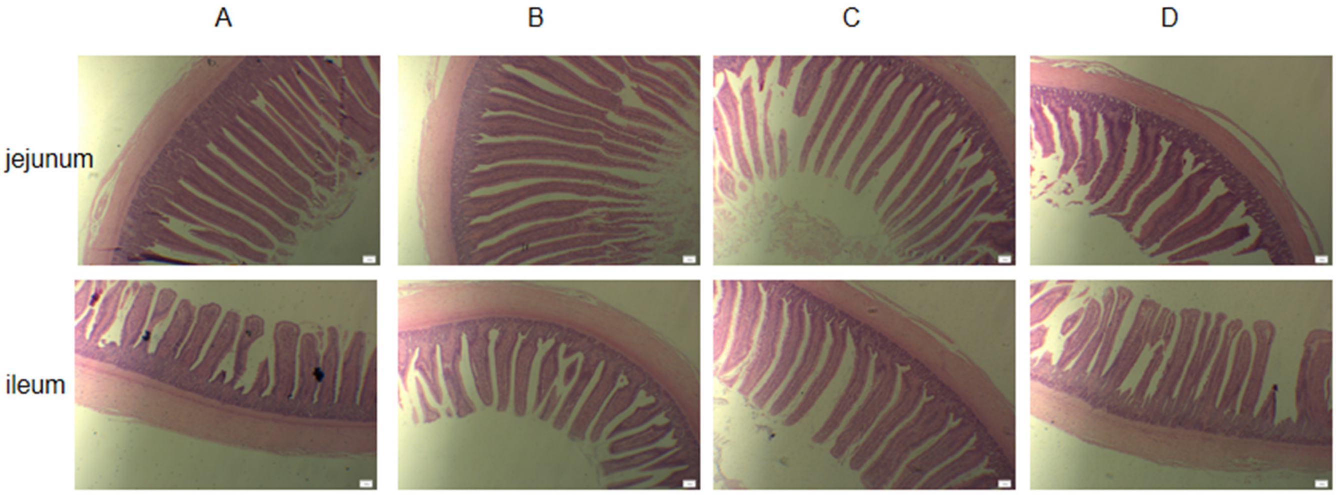

Figure S2. The representative images showing the effects of dietary probiotics on jejunal and ileal histomorphology of geese at the age of 70 days. Note: The group A as control was fed a basal diet, the treatment groups (B, C and D) were fed the basal diet supplemented with 250 mg/kg *Clostridium butyricum* (viable count was 3.0×10^6 CFU/g), 250 mg/kg *Bacillus subtilis* (viable count was 2.0×10^7 CFU/g), or the combination of 250 mg/kg *Clostridium butyricum* plus 250 mg/kg *Bacillus subtilis*, respectively.
